# The extent of physical and psychological workplace violence experienced by prehospital personnel in Denmark: a survey

**DOI:** 10.1186/s13049-024-01311-0

**Published:** 2024-12-23

**Authors:** Brit Schøsler, Frederik Stuhr Bang, Søren Mikkelsen

**Affiliations:** Prehospital Research Unit, Region of Southern Denmark, Odense, Denmark

**Keywords:** Prehospital exposure to violence, Prehospital harassment, Survey, Prehospital work environment

## Abstract

**Background:**

Workplace violence against healthcare workers has been a well-known problem for more than 40 years. This problem is also relevant for prehospital personnel who are at risk of physical and/or psychological violence during work. Violence and threats of violence can have physical and psychological consequences, including personal challenges in their everyday life, use of sick days, reports, and the need for professional help. Therefore, this study aimed to describe the extent of and subsequent reporting of physical and psychological workplace violence toward the prehospital healthcare workers in Denmark in a two-year period. Moreover, we wanted to elucidate any possible effect of workplace violence on the private and professional lives of the prehospital healthcare personnel.

**Methods:**

A nation-wide survey where a validated anonymised questionnaire was directed to all of the approximately 4500 Danish prehospital healthcare workers.

**Results:**

Out of 584 complete responses we found that 47.4% had experienced psychological violence on the job whereas 25.7% had experienced physical violence on the job within the past two years. The perpetrators were mainly patients or relatives of the patients. Physical violence was mostly reported as punching, pushing, and kicking, while psychological violence included threats of violence and other intimidation. After experiencing violence the respondents reported both physical and psychological harm, which for some prehospital healthcare workers had consequences for their professional and/or personal life. Furthermore, some prehospital healthcare workers reported that the violence had resulted in some patients receiving worse treatment afterwards. We found that violence was rarely reported to either employers or the police, because respondents believed the events were not important enough to merit reporting, or because a report was not considered to make any difference to the healthcare worker. The survey demonstrates that, as a minimum, at least one healthcare worker in 30 and one healthcare worker in 16 has been exposed to episodes of violence and threats of violence within the last two years.

**Conclusion:**

We suggest that the prehospital organisations emphasise reporting future episodes of physical and/or psychological violence. Knowing the extent of the problem is a prerequisite for addressing, debriefing, and/or other psychological follow-up.

**Trial registration:**

N/A.

**Supplementary Information:**

The online version contains supplementary material available at 10.1186/s13049-024-01311-0.

## Background

Workplace violence against healthcare workers (HCWs) has been a well-known problem for more than 40 years. Murray et al. found that the first peer-reviewed research on workplace violence against HCWs was published in 1993, but the issue was already discussed in industrial trade journals in 1978 [1, 2]. Data reveals that only 4% of HCWs in Europe have experienced physical or verbal workplace violence [3]. Especially emergency HCWs in the prehospital settings seem to experience a very high level of workplace violence, where one study even finds paramedics to have almost triple the odds of experiencing violence [4].

Workplace violence is a term that can vary in meaning between countries and objectivity from person to person. A clear definition is, therefore, important for a common understanding of the term. The World Health Organization generally defines workplace violence as incidents where staff are abused, threatened or assaulted in circumstances related to their work, involving an explicit or implicit challenge to their safety, well-being, or health [5]. For a deeper and more detailed understanding, workplace violence is grouped into physical and psychological violence. Physical violence includes kicking, punching, shoving, etc. Psychological violence includes verbal abuse, bullying/mobbing, harassment and threats [5].

Previous articles describing workplace violence against emergency HCWs has been hampered by not making sure there is a common understanding of physical and psychological violence by definitions to the respondents. It follows that a personal interpretation of workplace violence which may differ significantly. Furthermore, many studies measuring the frequency of workplace violence, do not group the term into physical and psychological violence. Therefore, a one-to-one comparison between articles on the matter can be difficult, as there are different definitions and understandings of workplace violence [2]. In addition, assessment of workplace violence in the prehospital setting has been conducted in various ways, the methods most used being cross-sectional surveys, direct observations, and injury reports. The variations between designs can make comparisons difficult [2].

Because of the rising focus on mental health and workplace environment, workplace violence is an important subject to highlight and closely monitor. So far, no Danish reports on workplace violence in the prehospital setting have been published. Therefore, the aim of this study is to describe physical and psychological workplace violence against the prehospital HCWs in Denmark in a two-year period. Our objective is to use a nation-wide survey to uncover the prevalence and the distribution of violence by demographic of the HCWs and perpetrators. Moreover, we want to show the effect it has on the private and professional lives of the prehospital healthcare personnel, and whether violence was reported to employers.

## Method

### System setting

Denmark is a Scandinavian country with a population of roughly 5.8 million people. All inhabitants have access to free healthcare. The emergency medical system provides services for patients without direct cost as it is a tax-funded three-tiered system [6]. The basic resource for simple emergencies is an ambulance manned by two emergency medical technicians (EMTs) and/or paramedics. In more complicated emergencies, an emergency medical dispatcher can dispatch an ambulance and a rapid response vehicle with a paramedic or an anaesthesiologist-manned mobile emergency care unit. Depending on the geographical location of the emergency the Helicopter Emergency Medical Services can be dispatched [6–8]. The prehospital findings and treatments are all registered in the nationwide prehospital medical records by the attending EMT, paramedic, or prehospital physician during and immediately after the treatment [7]. This allows for high quality population-based research and for improving the quality of care [6].

All Danish workers are protected by legislation concerning the work environment. In all the prehospital organisations, local representatives from both the employers and the employees are thus appointed to ensure a safe working environment. The local working environment organisation is obliged to offer assistance in connection with reporting to the police if an employee has been exposed to violence.

### Survey procedure

This study employed a nation-wide survey, which targeted the approximately 4500 prehospital personnel, including both prehospital physicians, paramedics, EMTs, and emergency dispatch personnel in all of the five healthcare regions of Denmark. The survey was developed in REDCap.

via the regional research service unit OPEN Patient Exploratory Network [9], and data were collected and managed using REDCap electronic data capture tools hosted at University of Southern Denmark. REDCap (Research Electronic Data Capture) is a secure, web-based software platform designed to support data capture for research studies, providing (1) an intuitive interface for validated data capture, (2) audit trails for tracking data manipulation and export procedures, (3) automated export procedures for seamless data downloads to common statistical packages, and (4) procedures for data integration and interoperability with external sources [10, 11].

The survey questions were validated by four lay persons and four healthcare professionals taking their comments into consideration in the final version of the survey questionnaire. Before distribution, the survey was pilot tested for design flaws. The survey was distributed by email to each healthcare region’s prehospital sector administrators, who then forwarded the survey to the employees. At the same time, the investigation was announced on various social media (LinkedIn, Facebook, Twitter). The survey informed the respondents that participation was voluntary, anonymous, and that answers would be confidential. The respondents were informed that consent to analyse the information given in the survey was assumed when the respondents filled out the questionnaire.

Three weeks after the initial survey distribution, a reminder was forwarded through the prehospital administrators in each of the five Danish health regions. The survey answers were collected from January 12th 2024, to April 1st 2024.

The survey questions were inspired by Lindquist et al. and adapted to the Danish prehospital scene [12]. As described in Murray et al., the definitions of physical and psychological violence are important to ensure a common understanding [2]. Therefore, these definitions were explained to the respondents of the survey.

The survey addressed three main areas concerning: Physical violence, psychological violence, and the demographic of the abused prehospital healthcare personnel.

The section on physical and psychological violence explored the amount and specifics of the violence, the abuser, the consequences of the physical or psychological abuse, and whether the police and/or the employer were notified of the incident.

The demographic section examined which healthcare region the respondents worked in, the work position, experience in that position, and the age and sex of the respondents. The survey can be found in Appendix 1 (Danish version) and Appendix 2 (English version).

### Statistical analyses

We used non-parametric statistics in the analyses. Descriptive statistics were used to report violence and age, gender, healthcare region, profession, and years of service.

STATA 18.0 (StataCorp, College Station, Texas, USA) was used for all analyses. The data were entered into an encrypted secure SharePoint hosted by the Region of Southern Denmark.

### Ethical approvals

All research was performed in accordance with all relevant national guidelines and regulations. The judicial office of the Odense University Hospital approved the data handling and storage. (Ref no. 23/55676). No further approvals are necessary in voluntary surveys according to Danish law [13]. All data handling was carried out respecting the Danish and European legislation concerning person-identifiable data [14, 15].

## Results

A total of 672 survey answers were collected amongst prehospital personnel in Denmark. In 88 of these, one or more of the sections of the survey were incomplete. These responses were excluded. 584 responses were thus processed. Therefore, an approximate response rate of 13.0% was recorded among the 4500 prehospital HCWs. In some of the multiple-choice questions during the survey, the respondents could give multiple answers, which explains why some numbers added up can be more than 100%.

The demographic characteristics data can be seen in Table 1. Most respondents were men (82.7%) between 30 and 49 years. There was an equal distribution of answers between the three largest healthcare regions: The Capital Region, the Region of Southern Denmark and the Central Denmark Region. Most of the respondents were emergency medical technicians (48.0%), paramedics (36.0%) or prehospital physicians (8.0%) and most had been in their profession for more than 10 years.

**Table 1:** Descriptive demographic characteristics of the respondents answering yes to stratified by physical violence and psychological violence from Danish study population of 584 individuals

	*N* (Physical violence)	*N* (Psychological violence)
**Age, years:**		
18–29	15	27
30–39	58	104
40–49	49	93
50–59	18	38
$\:\ge\:$60	10	15
**Sex:**		
Male	124	223
Female	25	53
Do not wish to answer	1	1
**Healthcare region:**		
The Capital Region	48	70
Region Zealand	10	24
Region of Southern Denmark	54	87
Central Denmark Region	28	65
North Denmark Region	10	31
**Profession:**		
Non-emergency patient transport personnel	5	7
EMTs	72	108
Paramedic	54	98
Prehospital physician	12	26
Emergency medical dispatcher	1	24
Technical dispatcher	0	1
Student	6	13
**Years in your profession:**		
0–1	4	7
2–4	14	36
5–9	35	57
$\:\ge\:\:$10	97	177

In the Danish survey in appendix 1 the profession category EMTs was separated in two categories: Ambulance attendant and ambulance assistant. These categories were merged afterwards because they have the same clinical assignments and fall under the same description in English as an EMT

An overview of the characteristics of the perpetrator and the specific act of physical or psychological violence experienced by respondents can be seen in Table 2. Within the past two years, 25.7% had been subjected to physical violence and 47.4% to psychological violence. Regarding physical violence, the majority who had experienced violence in the past two years had experienced it once or two to three times. The violence was mainly exerted by patients. The violence consisted mainly of striking with hand(s), pushing, kicking, or biting. Regarding psychological violence, the respondents had experienced violence once or two to three times in the past two years. The perpetrators were mainly patients or friends/family of the patient where male gender was overrepresented as perpetrator. The violence consisted of threats of physical violence, other intimidation, derogatory speech, or offensive speech/behavior. For details see Table 2.

**Table 2:** Characteristics of physical and psychological violence experienced by Danish prehospital personnel of 584 respondents

Physical violence	*N* (%)	Psychological violence	*N* (%)
**Experienced violence in the past 2 years:**		**Experienced violence in the past 2 years**:	
Yes	150 (25.7)	Yes	277 (47.4)
No	434 (74.3)	No	307 (52.6)
**How many episodes in the past 2 years** (*N* = 150):		**How many episodes in the past 2 years (***N*** = 277)**:	
1	79	1	72
2–3	67	2–3	139
4–6	3	4–6	38
≥ 7	1	≥ 7	28
**Who was the perpetrator** (*N*** = 150):**		**Who was the perpetrator** (*N*** = 277)**:	
Patient	148	Patient	243
Friend or family of the patient	26	Friend or family of the patient	130
Passer-by	4	Passer-by	37
Colleague	0	Colleague	13
Another HCWs	0	Another HCWs	16
House pet	11	Other	8
Other	0		
**Sex of perpetrator** (*N*** = 150):**		**Sex of perpetrator** (*N*** = 277)**:	
Male	111	Male	255
Female	76	Female	124
Do not wish to answer	0	Do not wish to answer	4
**What act of violence** (*N*** = 150):**		**What act of violence **(*N*** = 277)**:	
Strike with hand(s)	101	Threat of physical violence	197
Strike with an object	6	Other intimidation	114
Push	73	Derogatory speech	181
Kick	29	Offensive speech/behaviour	145
Strangulation	2	Teasing/bullying	19
Bite	16	Other	15
Shooting	1		
Stabbing	4		
Other	19		

It was possible to choose more than one answer in some of the questions. In these cases the summation of the answers can exceed the number of respondents

We found that a total of 10.0% of the respondents experiencing physical violence sustained physical injury, consisting mostly of wounds and/or bruises. It resulted in sick leave for 5.3% of those respondents. After experiencing physical violence 11.3% responded that the violence had consequences for their professional life mostly in the form of considering changing career and avoiding certain tasks. After experiencing physical violence, 6.7% responded the violence had consequences for their personal life, mostly in the form of disordered sleep. 34.7% answered that the patient received a worse treatment after a physically violent episode. The details for physical violence can be seen in Appendix 3, Supplemental Table 1.

We found that 6.5% of respondents experiencing psychological violence experienced mental injuries. The psychological injuries were mostly stress, anxiety and/or depression. It resulted in sick leave for 2.2% of those respondents. After experiencing psychological violence 15.2% expressed that the violence had consequences for their professional life. The respondents had mostly considered changing career and/or avoiding certain clinical tasks. After experiencing psychological violence 11.9% felt it had consequences for their personal life, mostly in the form of disordered sleep and/or other psychological symptoms. 29.2% answered that the patient received a worse treatment after a psychologically violent episode. The details for psychological violence can be seen in Appendix 3, Supplemental Table 2.

We found that 60.7% had not reported the physical violence to their employer and 62.0% had not reported the physical violence to the police. As for psychological violence we found that 83.0% of respondents did not report the violence to their employer and 83.4% did not report it to the police. The reason for not reporting the events for both physical and psychological violence were mainly because they did not find the episode(s) important enough, or because they did not believe that a report would make a difference. The details for not reporting episodes can be found in Table 3.

**Table 3:** Reasons for not reporting episodes of physical and psychological workplace violence to their employer and/or police of 584 respondents

Physical violence (*N*_Total_=150)	*N* (%)	Psychological violence (*N*_Total_=277)	*N* (%)
**Reported episode(s) as workplace injury:**		**Reported episode(s) as workplace injury**:	
Yes	59 (39.3)	Yes	47 (17.0)
No	91 (60.7)	No	230 (83.0)
**Why did you not report the episode(s)** (*N* = 91)		**Why did you not report the episode(s)** (*N*** = 230)**	
Fear of consequences of reporting	1	Fear of consequences of reporting	5
Fear of the perpetrator	0	Fear of the perpetrator	0
Shame	0	Shame	1
Did not find episode(s) important enough	53	Did not find episode(s) important enough	104
Did not believe a report would make a difference	34	Did not believe a report would make a difference	115
Other	17	Other	36
**Reported episode(s) to the police:**		**Reported episode(s) to the police**:	
Yes	57 (38.0)	Yes	46 (16.6)
No	93 (62.0)	No	231 (83.4)
**Why did you not report the episode(s)** (*N* = 93)		**Why did you not report the episode(s)** (*N*** = 231)**	
Fear of consequences of reporting	1	Fear of consequences of reporting	5
Fear of the perpetrator	1	Fear of the perpetrator	1
Shame	0	Shame	1
Did not find episode(s) important enough	54	Did not find episode(s) important enough	118
Did not believe a report would make a difference	33	Did not believe a report would make a difference	112
Other	16	Other	27

It was possible to choose all relevant answers in some of the questions. In these cases the summation of the answers can exceed the number of respondents

## Discussion

This study is the first in Denmark to investigate physical and psychological workplace violence against prehospital healthcare personnel. We found that almost half of the responding prehospital workers had been subjected to threats and/or other verbal abuse within the last two years, while almost one in four of the respondents had been physically abused within the last two years. Assuming that all prehospital HCW who did not answer the survey had never been exposed to physical violence or threats of violence, our results never the less are consistent with at least one prehospital HCW in 30 experiencing physical abuse and one in 16 experiencing verbal abuse within a two year period.

Other studies found that psychological violence made by patients was the most common form of violence, as reported on by the most recent studies [16, 17].

The prevalence of workplace violence is different, depending on the study and the country of origin. Denmark is rated as the second most peaceful country in the world [18], yet our data reveals workplace violence against prehospital HCWs is frequent. The psychological impact is significant. In one study, 94.1% stated that they at least once in their career had been subjected to abuse at work [16]. Another study of workplace violence among prehospital HCWs in India showed that the overall prevalence of physical and/or verbal assault was 67.9%. 56% reported that they were ‘somewhat worried’ about their overall safety, and 78.5% reported they had received no specific training on how to manage workplace violence [12].

Sahebi et al. have compared 14 studies focusing on workplace violence against HCWs in hospital and pre-hospital settings. The review concluded the overall prevalence of workplace violence is 58.7%. Of these, physical violence accounted for 20.8%, verbal violence for 66.8%, and sexual harassment for 10.5% [19].

Workplace violence can have severe repercussions. It has been shown to be directly related to burnout, lower job satisfaction, decreased work efficiency, less patient safety, and increased risk of mental illness [19, 20]. Savoy et al. reported a high number of minor psychological consequences some even with long-term impact [16].

Four Scandinavian studies of the general population investigated the effects that workplace violence and verbal abuse can have. Xu et al. reported that bullying and workplace violence can be a risk factor for type 2 diabetes mellitus [21]. Rudkjoebing et al. found an association between work-related threats or violence and an increased risk of depression two years later [22]. Furthermore, Hanson et al. observational data suggested that workplace violence is associated with an increased risk of suicide [23]. Lastly, Xu et al. concludes that bullying and violence are common at the workplace and that employees who are exposed to these stressors have a higher risk of cardiovascular disease [24].

With the high-risk environment, and the seemingly high prevalence among prehospital workers, several different strategies have been attempted to reduce workplace violence. Among these, zero-tolerance approaches and engaging with the perpetrator have been tested. Unfortunately, none of these strategies have been shown to reduce the workplace violence [4, 25].

The prehospital HCWs handles acute situations while surrounded by people who are often emotionally affected by the situation. Some of the respondents in our survey reported that physical and/or psychological violence against the prehospital HCWs could result in inferior treatment of the patient. Prehospital HCWs deals with daily ethical decision-making under time pressure, with limited information, and few colleagues to consult with in acute situations. Bruun et al. highlights an example where the HCWs were trying to save a severely bleeding patient while being threatened with death by the patient’s husband [26]. In similar situations, it seems almost impossible to continue work unaffected no matter the experience and skills of the HCWs.

Like our findings Mausz et al. found that less than 20% of the survey respondents had reported psychological workplace violence to the police or supervisors. Many respondents describe violence as ‘part of the job’ [27]. In Denmark, legislation concerning work environment stresses that reporting of episodes of violence should be aided by the employers. Emphasising the importance of reporting workplace violence to the police may help to address the extent of the phenomenon and may help ensure safety and aid in developing better preventive measures [27].

Future studies should focus on finding the right prevention against workplace violence. Starting by interviewing prehospital HCWs who already have experienced workplace violence. This is important to finding relevant solutions for the people dealing with this serious underlying problem.

### Strengths and limitations

This study has several strengths. Capturing the true prevalence of workplace violence may be difficult because of the individual understanding of violence; especially psychological violence. To overcome this problem, we stated the definition of physical and psychological violence when the respondents were asked if they had experienced workplace violence. Our survey was created as an anonymous survey accessible via a link or QR-code. The survey was distributed through the prehospital administrators as well as being announced on social media and by the use of “snowballing” where we encouraged the HCWs to re-distribute the survey among their colleagues. Via this method, we collected 584 relevant answers of the survey, which is a large absolute number of respondents compared to other similar studies like Savoy et al. with 273 respondents and Lindquist et al. with 386 respondents [12, 16]. We chose the public survey because it made it impossible to track the identity of the respondent, thus, maintaining the anonymity of the respondents. Keeping the survey anonymous was important because of the personal questions. We considered that it might be difficult for some people to answer honestly if the answers could be traced back to the individual.

The survey was voluntary with no consequences for not participating. The survey was addressed to all the relevant prehospital personnel in Denmark.

This study has some limitations. The first limitation is recall bias because the respondents were asked to remember two years back in time.

A second limitation is the need to limit the number of questions. Because of the time it takes to answer a survey, we chose fewer questions to ensure the respondents’ willingness to answer. Therefore, we deliberately did not include any questions about training or preventions of workplace violence.

Being a deliberate anonymous survey, it was impossible to perform a targeted follow-up. If we had made the respondents identifiable, we would have been able to send personalised reminders. Our study design did not allow for this.

Although the absolute number of respondents was high, an obvious potential limitation was the low response rate of 13.0%. The numbers presented thus represent the minimum number of episodes of physical and/or psychological violence.

## Conclusion

Even though Denmark is considered a safe country this study reveals that 47.4% of the responding prehospital workers had been subjected to threats and/or other verbal abuse, while 25.7% had been physically abused within the last two years. Even if one should consider that non-responders had not been exposed to physical or verbal abuse, this corresponds to one in 30 experiencing physical abuse and one in 16 experiencing verbal abuse within a two year period. This study reveals that the perpetrators are mainly patients or friend/family of the patients. Workplace violence can have severe consequences both professionally and personally for HCWs. Despite this, the violence is rarely reported to employer or police.

We therefore suggest the prehospital organisations emphasises reporting future episodes of physical and/or psychological violence. Knowing the extent of the problem is a prerequisite for addressing, debriefing, and/or other psychological follow-up.

## Electronic Supplementary Material

Below is the link to the electronic supplementary material.

Supplementary Material 1

Supplementary Material 2

## Data Availability

Anonymised data on the survey are available from the corresponding author on reasonable request.

## Abbreviations

HCWs: Healthcare workers
EMTs: Emergency medical technicians
REDCap: Research Electronic Data Capture
QR-code: Quick Response Code
