# Supplementary material for: The extent of physical and psychological workplace violence experienced by prehospital personnel in Denmark: a survey
Source: Scand J Trauma Resusc Emerg Med. 2024 Dec 23;32:136. doi: 10.1186/s13049-024-01311-0 (PMC11668072; doi:10.1186/s13049-024-01311-0)
Supplement: Supplementary file 1 — Supplementary Material 1 [file 13049_2024_1311_MOESM1_ESM.pdf]

Appendix 1 – Danish survey

Kære præhospitale medarbejder,
Formålet med undersøgelsen er at undersøge forekomsten og konsekvenserne af fysisk vold og psykisk vold (herunder trusler om fysisk vold) mod personale i den præhospitale enhed.
Besvarelserne skal bruges ifm. udarbejdelse af medicinsk kandidatspeciale, samt efterfølgende artikel.
Spørgeskemaet består af 3 sektioner, der omhandler 1) fysisk vold, 2) psykisk vold (herunder trusler om fysisk vold) og 3) anonyme data om dig som præhospital medarbejder.
Besvarelsen tager ca. 5-10 min.
Spørgeskemaet er anonymt, og det er frivilligt at deltage. Oplysningerne vil blive behandlet fortroligt. Når du indsender dine svar, giver du samtykke til, at dine svar må anvendes til at besvare ovenstående formål.
Ved spørgsmål, eller hvis du ønsker at trække dit samtykke tilbage, kan du kontakte projektmedarbejder og stud.med. Brit Schøsler på mail: Brit.Schosler@rsyd.dk.
Tak for hjælpen!

Fysisk vold

Har du været udsat for fysisk vold mod dig selv under udførelse af en ambulanceopgave, sygetransport eller arbejde i vagtcentralen inden for de sidste 2 år?	Yes<br>No<br><br>(Definition: Brug af fysisk vold mod dig. Resultatet kan medføre fysisk, seksuel eller psykisk skade. Det inkluderer bl.a. slag, spark, lussing, stik, skud, skub, bid (herunder af husdyr).)
Hvor mange hændelser af fysisk vold har du oplevet inden for de sidste 2 år?	1 gang<br>2-3 gange<br>4-6 gange<br>≥ 7 gange
Hvem har haft en fysisk voldelig opførsel overfor dig? (vælg alle relevante svar)	Patient<br>Pårørende<br>Forbipasserende<br>Kollega<br>Anden sundhedsmedarbejder<br>Husdyr<br>Andre
Hvad var kønnet på den/dem, som har udsat dig for fysisk vold? (vælg alle relevante svar)	Mand<br>Kvinde<br>Ønsker ikke at oplyse
Hvori bestod den fysiske vold? (vælg alle relevante svar)	Slag med hånd<br>Slag med genstand<br>Skub<br>Spark<br>Kvælertag / strangulering<br>Bid<br>Skud<br>Stikvåben<br>Andet
Fik du fysiske skader efter en eller flere hændelser?	Ja<br>Nej
Hvilke fysiske skader fik du? (vælg alle relevante svar)	Sår<br>Blå mærker<br>Hjernerystelse<br>Knoglebrud<br>Indre blødninger<br>Andet

Var du sygemeldt efter en eller flere hændelser?	Ja<br>Nej
Hvor mange dage var du i alt sygemeldt ca.?	______________________________________<br>(Ved sygemeldelse over flere perioder, skriv venligst samlet antal dage)
Har hændelsen/-erne haft konsekvenser for dig i dit erhvervsliv efterfølgende?	Ja<br>Nej
Hvorledes har hændelsen/-erne haft konsekvenser i dit erhvervsliv? (vælg alle relevante svar)	Overvejelse af skift af erhverv<br>Arbejdsopgaver du forsøger at undgå<br>Behandlingsforløb ved psykolog (stillet til rådighed af arbejdsgiver)<br>Fysisk skade som krævede lægelig behandling<br>Fysisk skade som IKKE krævede lægelig behandling<br>Andet<br>_____
Efter din vurdering, fik hændelsen/-erne indflydelse på din behandling af patienten?	Ingen betydning<br>Patienten fik dårligere behandling<br>Patienten fik bedre behandling<br>Ved ikke
Har hændelsen/-erne haft konsekvenser for dig i dit privatliv efterfølgende?	Ja<br>Nej
Hvorledes har hændelsen/-erne haft konsekvenser i dit privatliv? (vælg alle relevante svar)	Ændring i forholdet til din partner eller andre pårørende<br>Søvnproblemer<br>Andre psykiske symptomer f.eks. depression, stress eller selvmordstanker<br>Behandlingsforløb ved privat psykolog<br>Andet
Har du anmeldt en eller flere hændelser som en arbejdsskade?	Ja<br>Nej
Hvorfor anmeldte du ikke hændelsen/-erne? (vælg alle relevante svar)	Frygt for konsekvenserne ved anmeldelse<br>Frygt for voldspersonen<br>Skam over hændelsen<br>Hændelsen var ikke vigtig nok til en anmeldelse<br>En anmeldelse ville ikke gøre en forskel<br>Andet

Har du anmeldt en eller flere hændelser til politiet?	Ja<br>Nej
Hvorfor anmeldte du ikke hændelsen/-erne?<br>(vælg alle relevante svar)	Frygt for konsekvenserne ved anmeldelse<br>Frygt for voldspersonen<br>Skam over hændelsen<br>Hændelsen var ikke vigtig nok til en anmeldelse<br>En anmeldelse ville ikke gøre en forskel<br>Andet

Psykisk vold

Har du været udsat for psykisk vold eller trusler om fysisk vold mod dig selv under udførelse af en ambulanceopgave, sygetransport eller arbejde på vagtcentralen inden for de sidste 2 år?	Yes<br>No<br>(Definition: Bevidst brug af magt. Dette kan inkludere trusler om fysisk vold mod dig. Det inkluderer bl.a. verbalt misbrug, drilleri/mobning, chikane og trusler.)
Hvor mange hændelser af psykisk vold eller trusler om fysisk vold har du oplevet inden for de sidste 2 år?	1 gang<br>2-3 gange<br>4-6 gange<br>≥ 7 gange
Hvem har haft en psykisk voldelig opførsel over for dig? (vælg alle relevante svar)	Patient<br>Pårørende<br>Forbipasserende<br>Kollega<br>Anden sundhedsmedarbejder<br>Andre
Hvad var kønnet på den/dem, som har udsat dig for psykisk vold? (vælg alle relevante svar)	Mand<br>Kvinde<br>Ønsker ikke at oplyse
Hvori bestod den psykiske vold? (vælg alle relevante svar)	Trussel om fysisk vold<br>Anden intimidering<br>Nedværdigende tiltale<br>Krænkende ordvalg/adfærd<br>Drilleri/mobning<br>Andet
Fik du psykiske mén efter en eller flere hændelser?	Ja<br>Nej
Hvilke psykiske mén har du oplevet efter hændelsen/-erne? (vælg alle relevante svar)	Angst<br>Depression<br>Stress<br>PTSD<br>Selvmordstanker<br>Andet
Var du sygemeldt efter en eller flere hændelser?	Ja<br>Nej
Hvor mange dage var du ialt sygemeldt ca.?	______________________________<br>(Ved sygemeldelse over flere perioder, skriv venligst samlet antal dage)

Har hændelsen/-erne haft konsekvenser for dig i dit erhvervsliv efterfølgende?	Ja<br>Nej
Hvorledes har hændelsen/-erne haft konsekvenser for dig i dit erhvervsliv efterfølgende? (vælg alle relevante svar)	Overvejelse af skift af erhverv<br>Arbejdsopgaver du forsøger at undgå<br>Behandlingsforløb ved psykolog (stillet til rådighed af arbejdsgiver)<br>Andet
Efter din vurdering, fik hændelsen/-erne indflydelse på din behandling af patienten?	Ingen betydning<br>Patienten fik dårligere behandling<br>Patienten fik bedre behandling<br>Ved ikke
Har hændelsen/-erne haft konsekvenser for dig i dit privatliv efterfølgende?	Ja<br>Nej
Hvorledes har hændelsen/-erne haft konsekvenser for dig i dit privatliv efterfølgende? (vælg alle relevante svar)	Ændring i forholdet til din partner eller andre pårørende<br>Søvnproblemer<br>Andre psykiske symptomer f.eks. depression, stress eller selvmordstanker<br>Behandlingsforløb ved privat psykolog<br>Andet
Har du anmeldt en eller flere hændelser som en arbejdsskade?	Ja<br>Nej
Hvorfor anmeldte du ikke hændelsen/-erne? (vælg alle relevante svar)	Frygt for konsekvenserne ved anmeldelse<br>Frygt for voldspersonen<br>Skam over hændelsen<br>Hændelsen var ikke vigtig nok til en anmeldelse<br>En anmeldelse ville ikke gøre en forskel<br>Andet
Har du anmeldt en eller flere hændelser til politiet?	Ja<br>Nej
Hvorfor anmeldte du ikke hændelsen/-erne? (vælg alle relevante svar)	Frygt for konsekvenserne ved anmeldelse<br>Frygt for voldspersonen<br>Skam over hændelsen<br>Hændelsen var ikke vigtig nok til en anmeldelse<br>En anmeldelse ville ikke gøre en forskel<br>Andet

Demografi

Hvilken region arbejder du primært i?	Region Hovedstaden<br>Region Sjælland<br>Region Syddanmark<br>Region Midtjylland<br>Region Nordjylland
Hvilken stilling har du?	Sygetransportchauffør / ST-redder<br>Ambulanceassistent<br>Ambulancebehandler<br>Paramediciner<br>Præhospital læge<br>Sundhedsfaglig visitator<br>Teknisk disponent i vagtcentral<br>Elev
Hvor lang tid har du været i tjenesten?	0 – 1 år<br>2 - 4 år<br>5 - 9 år<br>10+ år
Hvad er din alder?	18-29 år<br>30-39 år<br>40-49 år<br>50-59 år<br>≥ 60 år
Hvilket køn har du?	Mand<br>Kvinde<br>Ønsker ikke at oplyse

Fremtidige interviews

Må vi kontakte dig fremover, hvis der laves yderligere undersøgelser på området, f.eks. interviews ifm. fysisk eller psykisk vold mod præhospitalt personale?<br>Hvis du er interesseret i dette, beder vi dig om at udfylde navn og e-mail. Uanset om du svarer ja eller nej, vil informationerne fra dette spørgeskema behandles anonymt. Ja Nej	Ja - Yes<br>Nej - No

Tak fordi du er interesseret i at medvirke i evt. yderligere undersøgelser på området, f.eks. interviews ifm. fysisk eller psykisk vold mod præhospitalt personale.

Udfyld venligst navn og e-mail nedenfor

Dit fulde navn ____________

Din e-mail _______________

Appendix 2 – English survey

Dear prehospital personnel,
The purpose of this survey is to uncover the frequency and consequences of physical and phycological violence targeted at prehospital personnel. The answers are used for a dissertation in Medicine and an article on the matter.
The survey has three sections: 1) Physical violence; 2) phycological violence (including threats of violence); 3) anonymous data about you as an employee in a prehospital field.
We estimate that it will take 5-10 minutes to fill out the survey.
The survey is anonymized, and participation is voluntary. The data is handled with great confidentiality. With your consent, your answers will be used for the abovementioned purposes.
If you have questions or want to withdraw your consent, you can contact project manager and student of medicine, Brit Schøsler via mail: brit.schoesler@rsyd.dk.
Thank you very much!

Physical violence

Have you experienced physical violence target at you while on duty within the past 2 years?	Yes<br>No<br><br>(Definition: The use of physical force against another person or group, that results in physical, sexual, or psychological harm. It includes among others, beating, kicking, slapping, stabbing, shooting, pushing, biting and pinching.)
How many episodes of physical violence have you experienced within the past 2 years?	1<br>2-3<br>4-6<br>≥ 7
Who was the perpetrator of physical violence against you? (choose all relevant answers)	Patient<br>A friend or family member of the patient<br>Passer-by<br>Colleague<br>Another healthcare worker<br>House pet<br>Other
What was the sex of the person(s) who committed an act of physical violence against you? (choose all relevant answers)	Male<br>Female<br>I do not wish to answer
What was the act of violence? (choose all relevant answers)	Strike with hand(s)<br>Strike with an object<br>Shove<br>Kick<br>Choke hold<br>Bite<br>Shot at with a firearm<br>Bladed weapon<br>Other
Did you sustain any physical injuries?	Yes<br>No
What kind of physical injury? (choose all relevant answers)	Wound<br>Bruising<br>Concussion<br>Broken bone(s)<br>Internal bleeding<br>Other

Did you get sick leave or called in sick after any of the episodes of violence?	Yes<br>No
How many sick days did you have in total?	______________________________________<br>(If you have had time off several times, please state the total number of days)
Did the episode(s) have any consequences for your professional life afterward?	Yes<br>No
How did the episode(s) change your professional life? (choose all relevant answers)	Considered changing career<br>Tend to avoid certain clinical tasks<br>Received treatment from a mental health professional (provided by my employer)<br>Sustained physical injuries which required medical care<br>Sustained physical injuries which did not require medical care<br>Other
How did the episode(s) affect your treatment of the patient?	No change<br>The patient received a worse treatment than normal<br>The patient received a better treatment than normal<br>I do not know
Did the episodes(s) have any consequences for your personal life afterward?	Yes<br>No
How did the episode(s) change your personal life? (choose all relevant answers)	Changed my relationship to my partner or friends/family<br>Disordered sleep<br>Other phycological symptoms, such as: Depression, stress, suicidal thoughts<br>Received treatment from a mental health professional (sought privately)<br>Other
Have you reported the episode(s) as a workplace injury?	Yes<br>No
Why did you not report the episode(s)? (choose all relevant answers)	Fear of the consequences of reporting<br>Fear of the perpetrator<br>Shame<br>I did not find the episode(s) important enough to report

	I did not believe that a report would make a difference<br>Other
Have you reported the episode(s) to the police?	Yes<br>No
Why did you not report the episode(s)?<br>(choose all relevant answers)	Fear of the consequences of a report<br>Fear of the perpetrator<br>Shame<br>I did not find the episode(s) important enough to report<br>I did not believe that a report would make a difference<br>Other

Psychological violence

Have you experienced psychological violence or threats of violence target at you while on duty within the past 2 years?	Yes<br>No<br><br>(Definition: Intentional use of power, including threat of physical force, against another person or group, that can result in harm to physical, mental, spiritual, moral, or social development. It includes verbal abuse, bullying/mobbing, harassment and threats.)
How many episodes of psychological violence or threats of violence have you experienced within the past 2 years?	1<br>2-3<br>4-6<br>≥ 7
Who was the perpetrator of psychological violence or threat of violence against you? (choose all relevant answers)	Patient<br>Friend or family member of the patient.<br>Passer-by<br>Colleague<br>Another health care worker.<br>Other
What was the sex of the person(s) who committed an act of psychological violence against you? (choose all relevant answers)	Male<br>Female<br>I do not wish to answer
What was the act of the psychological violence? (choose all relevant answers)	Threat of physical violence<br>Other intimidation<br>Derogatory speech<br>Offensive speech/behaviour<br>Teasing/bullying<br>Other
Did you sustain any mental injuries?	Yes<br>No
What kind of mental injury? (choose all relevant answers)	Anxiety<br>Depression<br>Stress<br>PTSD<br>Suicidal thoughts<br>Other
Did you get sick leave or called in sick after any of the episodes of violence?	Yes<br>No

How many sick days did you have in total?	______________________________<br>(If you have had time off several times, please state the total number of days.)
Did the episode(s) have any consequences for your professional life afterward?	Yes<br>No
How did the episode(s) change your professional life? (choose all relevant answers)	Considered changing career<br>I tend to avoid certain clinical tasks<br>Received treatment from a mental health professional (provided by my employer)<br>Other
How did the episode(s) affect your treatment of the patient?	No chance<br>The patient received a worse treatment than normal<br>The patient received a better treatment than normal<br>I do not know
Did the episodes(s) have any consequences for your personal life afterward?	Yes<br>No
How did the episode(s) change your personal life? (choose all relevant answers)	Changed my relationship to patients and their friends/family<br>Disordered sleep<br>Other psychological symptoms, such as: Depression, stress, or suicidal thoughts<br>Received treatment from a mental health professional (sought privately)<br>Other
Have you reported the episode(s) as a workplace injury?	Yes<br>No
Why did you not report the episode(s)? (choose all relevant answers)	Fear of the consequences of reporting<br>Fear of the perpetrator<br>Shame<br>I did not find the episode(s) important enough to report<br>I did not believe that a report would make a difference<br>Other
Have you reported the episode(s) to the police?	Yes<br>No
Why did you not report the episode(s)? (choose all relevant answers)	Fear of the consequences of reporting<br>Fear of the perpetrator

	Shame<br>I did not find the episode(s) important enough to report<br>I did not believe that a report would make a difference<br>Other

Demographics

What region do you primarily work in?	The Capital Region<br>Region Zealand<br>Region of Southern Denmark<br>Central Denmark Region<br>North Denmark Region
What is your profession?	Non-emergency patient transport personnel<br>Emergency Medical Technician<br>Paramedic<br>Prehospital physician<br>Emergency medical dispatcher<br>Technical dispatcher<br>Student
How long have you been in that profession?	0 - 1 year<br>2 - 4 years<br>5 - 9 years<br>10+ years
What is your age?	18-29 years old<br>30-39 years old<br>40-49 years old<br>50-59 years old<br>≥ 60 years old
What is your sex?	Male<br>Female<br>I do not wish to answer

Future interviews

Can we contact you in the future if further studies are carried out in the area, e.g. interviews related to physical or psychological violence against prehospital personnel?<br>If you are interested, please fill out your name and email. Regardless of whether you answer yes or no, the information from this questionnaire will be treated anonymously.	Yes<br><br>No

Thank you for your interest in participating in further studies on this subject, e.g. interviews focusing on physical or psychological violence against prehospital personnel.

Please, fill out name and e-mail below:

Your full name ____________

Your e-mail _______________
