# Supplementary material for: The extent of physical and psychological workplace violence experienced by prehospital personnel in Denmark: a survey
Source: Scand J Trauma Resusc Emerg Med. 2024 Dec 23;32:136. doi: 10.1186/s13049-024-01311-0 (PMC11668072; doi:10.1186/s13049-024-01311-0)
Supplement: Supplementary file 2 — Supplementary Material 2 [file 13049_2024_1311_MOESM2_ESM.docx]

| **Supplemental Table 1: Consequences experienced after physical violence answered by 150 respondents.** | | | |
| --- | --- | --- | --- |
|  | **N (%)** |  | **N (%)** |
| **Did you sustain any physical injuries:** |  | **How did episode(s) change your professional life:** |  |
| Yes | 15 (10.0) | Considered changing career | 4 |
| No | 135 (90.0) | Tend to avoid certain tasks | 7 |
| **What kind of physical injury:** |  | Treatment from mental health professional | 2 |
| Wound | 8 | Physical injury required medical treatment | 1 |
| Bruising | 9 | Physical injury did not require medical treatment | 1 |
| Concussion | 0 | Other | 7 |
| Broken bone(s) | 1 | **Consequences for your personal life afterward:** |  |
| Internal bleeding | 1 | Yes | 10 |
| Other | 1 | No | 140 |
| **Did you get sick leave/called in sick after any episodes:** |  | **How did episode(s) change your personal life:** |  |
| Yes | 8 | Changed my relationship to my partner or friends/family | 2 |
| No | 142 | Disordered sleep | 8 |
| **How many sick days:** |  | Other psychological symptoms such as: Depression, stress, suicidal thoughts | 3 |
| Total amount from events (days, mean) | 14.5 | Treatment from mental health professional | 2 |
| **Consequences for your professional life afterward:** |  | Other | 2 |
| Yes | 17 | **How did the episode(s) affect your treatment of the patient:** |  |
| No | 133 | No change | 84 |
|  |  | Patient received a worse treatment | 52 |
|  |  | Patient received a better treatment | 1 |
|  |  | Do not know | 13 |

It was possible to choose all relevant answers in some of the questions. In these cases, the summation of the answers can exceed the number of respondents.

| Supplemental Table 2: Consequences experienced after psychological violence answered by 277 respondents. | | | |
| --- | --- | --- | --- |
|  | **N (%)** |  | **N (%)** |
| Did you sustain any mental injuries: |  | **How did episode(s) change your professional life:** |  |
| Yes | 18 (6.5) | Considered changing career | 20 |
| No | 259 (93.5) | Tend to avoid certain clinical tasks | 20 |
| What kind of mental injury: |  | Treatment from mental health professional | 7 |
| Stress | 7 | Other | 13 |
| Anxiety | 7 | **Consequences for your personal life afterward:** |  |
| Depression | 4 | Yes | 33 |
| PTSD | 3 | No | 244 |
| Suicidal thoughts | 1 | **How did episode(s) change your personal life:** |  |
| Other | 4 | Changed my relationship to my partner or friends/family | 9 |
| Did you get sick leave/called in sick after any episodes: |  | Disordered sleep | 18 |
| Yes | 6 | Other psychological symptoms such as: Depression, stress, suicidal thoughts | 12 |
| No | 271 | Treatment from mental health professional | 5 |
| How many days of sick leave: |  | Other | 9 |
| Total amount from events (days, mean) | 47.5 | **How did the episode(s) affect your treatment of the patient:** |  |
| Consequences for your professional life afterward: |  | No change | 158 |
| Yes | 42 | Patient received a worse treatment | 81 |
| No | 235 | Patient received a better treatment | 4 |
|  |  | Do not know | 34 |

It was possible to choose all relevant answers in some of the questions. In these cases, the summation of the answers can exceed the number of respondents.
